# Friend of GATA (FOG) Interacts with the Nucleosome Remodeling and Deacetylase Complex (NuRD) to Support Primitive Erythropoiesis in *Xenopus laevis*

**DOI:** 10.1371/journal.pone.0029882

**Published:** 2012-01-03

**Authors:** Mizuho S. Mimoto, Jan L. Christian

**Affiliations:** Department of Cell and Developmental Biology, School of Medicine, Oregon Health and Science University, Portland, Oregon, United States of America; Radboud University Nijmegen, The Netherlands

## Abstract

Friend of GATA (FOG) plays many diverse roles in adult and embryonic hematopoiesis, however the mechanisms by which it functions and the roles of potential interaction partners are not completely understood. Previous work has shown that overexpression of FOG in *Xenopus laevis* causes loss of blood suggesting that in contrast to its role in mammals, FOG might normally function to repress erythropoiesis in this species. Using loss-of-function analysis, we demonstrate that FOG is essential to support primitive red blood cell (RBC) development in *Xenopus*. Moreover, we show that it is specifically required to prevent excess apoptosis of circulating primitive RBCs and that in the absence of FOG, the pro-apoptotic gene *Bim-1* is strongly upregulated. To identify domains of FOG that are essential for blood development and, conversely, to begin to understand the mechanism by which overexpressed FOG represses primitive erythropoiesis, we asked whether FOG mutants that are unable to interact with known co-factors retain their ability to rescue blood formation in FOG morphants and whether they repress erythropoiesis when overexpressed in wild type embryos. We find that interaction of FOG with the Nucleosome Remodeling and Deacetylase complex (NuRD), but not with C-terminal Binding Protein, is essential for normal primitive RBC development. In contrast, overexpression of all mutant and wild type constructs causes a comparable repression of primitive erythropoiesis. Together, our data suggest that a requirement for FOG and its interaction with NuRD during primitive erythropoiesis are conserved in *Xenopus* and that loss of blood upon FOG overexpression is due to a dominant-interfering effect.

## Introduction

Vertebrate blood development takes place in two waves, referred to as primitive and definitive hematopoiesis. Primitive hematopoiesis is the initial wave of blood development in the embryo in which blood progenitor cells in the mesoderm give rise mainly to primitive red blood cells (RBCs), although some white blood cells are also produced at this time. By comparison, definitive hematopoiesis constitutes the second wave of blood development in which hematopoietic stem cells give rise to blood cells of all lineages (reviewed in [1] and [2]). Definitive hematopoiesis begins later in development and continues throughout adult life. In mammalian embryos primitive hematopoiesis takes place extra-embryonically in the yolk sac blood islands [2]. The analogous structure in *Xenopus* embryos is called the ventral blood island (VBI). The VBI is an intraembryonic structure that forms when distinct populations of cells from the rostral and caudal mesoderm converge during gastrulation and subsequently elongate along the ventral midline [1], [3]. As RBCs form during the early tailbud stage, they begin to express the differentiation marker *globin*, which initiates in the anterior aspect of the VBI and extends posteriorly as more hemoglobin is synthesized. During the late tailbud stage (roughly stage 35–36), the heart begins to contract and RBCs begin circulating within the embryo [4].

The GATA family of zinc finger transcription factors plays an essential role during hematopoiesis. There are six vertebrate GATAs; GATA-1, -2 and -3 are required for hematopoiesis, whereas GATA-4, -5, and -6 are required for cardiac, endoderm, gonadal and CNS patterning [5], [6] (and reviewed in [7]). Targeted deletion of *GATA-1* causes mice to die early in embryonic development from severe anemia [5], [6]. Specifically, *GATA-1* null embryos have defects in primitive erythropoiesis, and RBC progenitor development is arrested at the proerythroblast stage [5]. Similarly, zebrafish embryos in which GATA-1 is either mutated or depleted with antisense morpholino oligonucleotides (MOs) lack RBCs [8], [9]. GATA-1 has also been shown to support the viability of RBC precursors *in vitro* by suppressing apoptosis [10].

FOG-1 (Friend of GATA 1) functions as a critical transcriptional cofactor for both GATA-1 and GATA-2 during hematopoiesis. FOG is a large multi-domain protein that includes nine conserved zinc fingers, four of which mediate GATA binding [11], [12]. In the mouse, targeted deletion of FOG-1 blocks primitive erythropoiesis at the pro-erythroblast stage, phenocopying the RBC defect seen in *GATA-1* null mice [13]. This function appears to be conserved in zebrafish, as RBCs from embryos in which FOG has been depleted are properly specified but fail to mature [14]. Interestingly, point mutations in humans that disrupt GATA-1/FOG-1 interaction are associated with familial dyserythropoietic anemia and thrombocytopenia [15], and a point mutation in GATA-1 that inhibits FOG-1 binding cannot rescue erythroid differentiation in a GATA-1 deficient cell line. This defect, however, is rescued by co-expression of FOG-1 that bears a reciprocal mutation that restores binding [16]. The similarity in the *FOG-1* and *GATA-1* loss-of-function phenotypes together with *in vitro* mutant rescue analysis strongly suggest that they function in concert to promote RBC development.

There are two FOG homologs in the mouse, *mFOG-1* and *mFOG-2*. Although they appear to be functionally redundant (e.g. forced expression of FOG-2 can rescue erythroid maturation in a *FOG-1^-/-^* cell line [17]) their non-overlapping patterns of expression enforce interaction with different GATA subfamilies, and result in distinct *in vivo* functions. Specifically, FOG*-*1 interacts with GATA-1, -2 and -3 and is involved in hematopoiesis, while FOG-2 interacts primarily with GATA-4, -5 and -6 (reviewed in [18]) to regulate development of other organ systems. FOG is both a transcriptional co-activator and co-repressor of GATA target genes [19], [20], [21], [22], but the mechanisms by which it carries out each of these functions is unclear. In addition to its interaction with GATA, FOG also has been shown to recruit C-terminal binding protein (CtBP) and members of the Nucleosome Remodeling and Deacetylase (NuRD) complex. Binding domains for CtBP and NuRD are highly conserved across species in the majority of FOG isoforms [23], [24], [25]. Both are ubiquitously expressed during early *Xenopus* development [26], [27], [28], and would thus be spatially and temporally poised to function in primitive erythropoiesis.

While an essential role for FOG-1 during hematopoiesis has been well documented in mice and fish, the role of *Xenopus* FOG (xFOG) in this process is less clear. Only one FOG homolog has been identified in *Xenopus laevis* (Figure S1 and [29]). It is most similar to mFOG-1 but is co-expressed with, and thus predicted to interact with all six GATA factors [29]. The current model of FOG function in frogs is based primarily on overexpression of either a truncated *Xenopus* transcript or mouse FOG-2 (mFOG-2) in *Xenopus* embryos, both of which led to defects in primitive erythropoiesis [29]. Additionally these studies demonstrated that overexpression of an mFOG-2 mutant that was unable to bind CtBP did not inhibit blood formation and in fact led to greater hematopoietic activity. These findings suggest that in frogs, xFOG is acting solely as a transcriptional repressor through recruitment of CtBP, and that its normal function is to inhibit blood development [29]. This interpretation would suggest that FOG function in *Xenopus* is significantly different from its role in mice and fish.

Mice that harbor a targeted knock-in allele of FOG that lacks CtBP binding capacity show no defects in hematopoiesis, suggesting that this domain may be dispensable for normal function [23]. By contrast, mice that harbor a targeted knock-in allele of FOG that lacks NuRD binding capacity exhibit defects in fetal hepatic and adult marrow-derived definitive erythropoiesis and megakaryopoiesis [30], [31]. A subset of these mutant embryos have pale yolk sacs at E10.5–12.5 suggesting that primitive hematopoiesis might also be impaired [31]. However, most homozygous mutants appear indistinguishable from controls at E12.5 and most survive to adulthood, indicating that primitive hematopoiesis is not completely absent [31]. The question as to how loss of FOG-NuRD interaction affects primitive erythropoiesis *in vivo* is thus still open.

In the current study, we have used a loss of function approach to address discrepancies in the literature regarding FOG function during primitive hematopoiesis in *Xenopus*. We find that xFOG and its interaction with the NuRD complex are required for primitive erythropoiesis, but that direct interaction with CtBP is dispensable. While we find that overexpression of xFOG also disrupts normal blood development, we show that it does so independent of its ability to interact with other cofactors known to be required for blood. This phenomenon is thus more consistent with a dominant-negative effect of xFOG overexpression rather than a reflection of its endogenous function. Finally, we show that loss of xFOG leads to upregulation of the pro-apoptotic gene *Bim-1* and excessive apoptosis of RBCs. Taken together, these studies show that FOG function is in fact conserved in *Xenopus* and is important for primitive RBC survival.

## Materials and Methods

### Ethics Statement

This study was carried out in strict accordance with the recommendations in the Guide for the Care and Use of Laboratory Animals of the National Institutes of Health. The protocol was approved by the Institutional Animal Care and Use Committee of Oregon Health and Science University (Protocol number A719).

### Embryo culture and manipulation

Ovulation was induced in adult *Xenopus laevis* females by injection of 50 IU of human chorionic gonadotropin (Sigma) into the dorsal lymph sac to induce spawning the night before egg collection. Embryos were staged according to Nieuwkoop and Faber [32], [33]. Capped synthetic mRNA was synthesized by *in vitro* transcription of linearized template cDNA using a MegaScript kit (Ambion) and injected into the two vegetal blastomeres on the ventral side of eight-cell embryos to target prospective blood forming cells, as described previously [33].

### Morpholinos and cDNA constructs

Sequence encoding the N-terminus of *Xenopus FOG* was obtained by 5′ RACE using oligonucleotide primers complementary to sequence near the 5′ end of the published partial length *xFOG* cDNA (accession no. AF241228). RNA was isolated from *Xenopus* embryos at stage 34, reverse transcribed and used as a template for PCR-mediated amplification of an *xFOG* cDNA (GenBank accession number GU384581) that encodes the entire open reading frame, as determined by alignment with *FOG* from other species (Figure S1). A closely related cDNA that most likely represents a duplicate copy of the *xFOG* gene was identified by searching The Gene Index Project database (Accession number TC411807). Morpholino antisense oligonucleotides (MOs) complimentary to both alleles of *xFOG* (xFOGa: 5′-ATTGCTCTGTTTCCTTCTGGACATG-3′ and xFOGb: GCTGGAGGACAAGGCAGGATCAAGC) were purchased from Gene Tools, LLC (Philomath, OR). Equal amounts of the two FOG MOs were mixed and the dose was titrated to 40 ng per embryo. Sequence encoding a MYC epitope tag was appended to the 5′ end of the *xFOG* open reading frame by PCR-mediated amplification. Silent mutations to prevent morpholino annealing were engineered using a QuikChange XL (Stratagene) mutagenesis kit to generate a FOG rescue construct (xFOGr) with the following sequence at the 5′ end: 5′*ATG* **GAA CAA AAA CTT ATT TCT GAA GAA GAT CTG** TC***T*** AGA ***C***GG AA***G*** CAG AG***T*** AA***C*** CCC AGA CAG. MYC tag is in bold, morpholino 2 target sequence is underlined, translation start site is italicized and silent mutations are bold and italicized. xFOG or xFOGr was used as a template to generate cDNAs encoding xFOGΔCtBP and xFOGΔNuRD by PCR-mediated mutagenesis. xFOGΔCtBP has a two-residue substitution that converts the CtBP binding motif from PIDLSK to PIASSK. This mutation has been shown to disrupt binding of CtBP to mouse FOG-2 [29] and FOG-1 [23]. xFOGΔNuRD lacks amino acids two through twelve, which have been shown to be essential for NuRD binding [34]. The xFOGΔNuRD/ΔCtBP double mutant was generated by subcloning the CtBP substitution into the xFOGΔNuRD construct using restriction enzymes. xFOG4ZM was generated by using a QuikChange XL mutagenesis kit (Stratagene). xFOG4ZM harbors a single tyrosine to alanine substitution in each of the four GATA-binding zinc fingers (fingers 1,5,6 and 9) [12]. Introduction of analogous point mutations into mouse FOG-1 has been shown to be sufficient to inhibit GATA-1 binding [35]. All cDNAs were subcloned into pCS2+ for RNA transcription and transient transfection of mammalian cells.

### Analysis of RNA

Total RNA was isolated and Northern blots were hybridized with antisense riboprobes as described previously [36], [37]. Bands were visualized with a phosphoimager and quantified using the NIH ImageJ software. Embryos were processed for *in situ* hybridization according to the protocol outlined in [38]. For Quantitative Real Time PCR (qPCR), total RNA was isolated using Trizol reagent per the manufacturer's protocol (Invitrogen) and purified using an RNeasy spin column (QIAGEN). For each sample, 2.5 µg of purified RNA was used as template for first strand cDNA synthesis with random oligo dT primers and SuperScript III reverse transcriptase (Invitrogen) per the manufacturer's protocol. Transcript levels were evaluated by qRT-PCR using the SYBR Green reagent (Invitrogen) on an ABI 7900HT with the following reaction conditions: 95°C, 10 minutes, followed by 95°C, 15 seconds and 60°C for 1 minute for 40 cycles. Expression levels were calculated based on standard curves generated from serial dilutions of cDNA from each sample. All samples were analyzed in triplicate and normalized to ODC. Primers used are as follows: *ODC*: F 5′-TGC AGA GCC TGG GAG ATA CT-3′, R 5′-CAT TGG CAG CAT CTT CTT CA-3′; *GATA-*1: F 5′-GGA GAC GGA ATG CAA GTG GAG AC-3′, R 5′- CCT GCT GCT CAC CTT TCG GTT C-3′ *Bim1*: F 5′-CAG AAC TGT GGA TAG CAC AGG AAC-3′, R 5′- CAG ACA AGC TGA GTC ACT TCT CG-3′.

### Immunoprecipitation and Western blot analysis

xFOG and xFOG mutant expression vectors were generated by subcloning full-length *Xenopus* FOG into pCS2^+^. To verify that FOG MO targets xFOG but not xFOGr, 0.5 µg of each construct was injected into *Xenopus* embryos at the 2–4 cell stage. Embryos were cultured to stage 15 and protein was harvested from by freon extraction as described previously [36]. Proteins were denatured by boiling in loading buffer with 8% SDS and 5% β−mercaptoethanol, separated by 10% SDS-PAGE and transferred onto PVDF membrane. Membranes were probed with mouse 9E10 anti-MYC (1∶500) and anti-actin (Sigma, 1∶10,000) antibodies overnight at 4°C detected by chemiluminescence (Pierce).

The xGATA-2HA expression vector has been described previously [39]. HeLa cells were transfected with 0.5 µg xGATA-2HA and 5.0 µg wild type or mutant xFOG-MYC using lipofectamine 2000 (Invitrogen) as per the manufacturer's protocol. Cells were lysed after 24 hours and protein extracts prepared as described previously [40]. Protein extracts were incubated in 750 µL binding buffer [41] containing 1∶500 anti-MYC antibody (9B11; Sigma) at 4°C overnight. Complexes were precipitated with Protein G conjugated sepharose beads (Invitrogen) at 4°C for three hours, then washed four times in binding buffer. Proteins were denatured by boiling in loading buffer with 8% SDS and 5% β−mercaptoethanol, separated by 7% or 8% SDS-PAGE and transferred onto PVDF membrane. Membranes were probed with anti-HA (3F10; Roche, 1∶1000), anti-MYC (71D10; Cell Signaling, 1∶500) and anti-actin (Sigma, 1∶10,000) antibodies and detected by chemiluminescence (Pierce).

### Collection and analysis of peripheral blood samples

Tails were severed from tadpoles and blood was collected into medium containing 0.7X PBS, 0.5% BSA and 10 IU/ml of heparin. Cells were concentrated onto slides using a cytospin and stained with the Hema 3 stain set (Fisher Diagnostics). A minimum of 20 embryos were bled per experimental group and for each embryo the number of cells present in four random fields was counted at 20X magnification. Each experiment was repeated a minimum of three times and results were pooled.

### Tunel

Blood from stage 42 embryos was collected onto slides as described above and assessed for apoptosis using the DeadEnd Fluorometric TUNEL kit (Promega). Nuclei of cells were counterstained with propidium iodide to determine total numbers of cells. A minimum of 20 embryos were bled per experimental group and for each embryo the number of apoptotic and total cells present in four random fields was counted at 20X magnification. Each experiment was repeated a minimum of three times and results were pooled.

## Results and Discussion

### FOG is required for primitive red blood cell development in Xenopus

**Figure 1:**
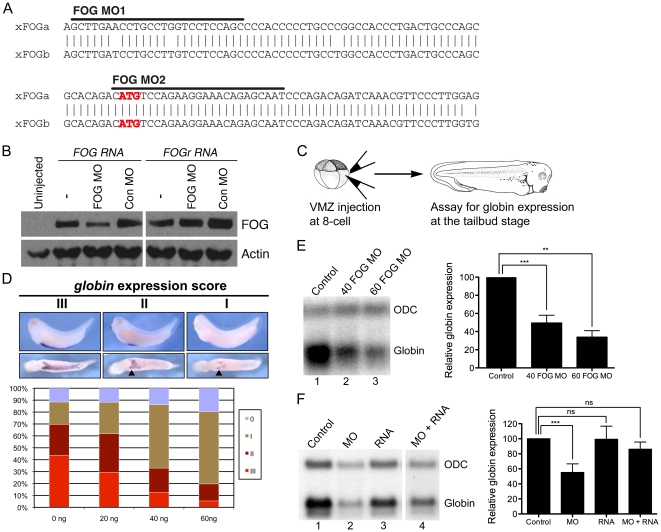
FOG is required for primitive erythropoiesis in *Xenopus laevis*. (A) Alignment of sequence surrounding the translation start site of the two *Xenopus FOG* alleles. Sequences to which FOG MOs bind are indicated by the black bars and the ATG start codon is in red. (B) Western blot of lysates from embryos injected with MYC epitope tagged wild type xFOG or xFOGr RNA, which harbors silent mutations that prevent MO binding, with either FOG MO or Control MO. Note that FOG MOs inhibit the translation of xFOG-MYC RNA but do not affect translation of xFOGr-MYC RNA. Actin is shown as a loading control. (*n = 3*) (C) Schematic of experimental design. MO and/or RNA is targeted to the ventral marginal zone (VMZ) of an eight-cell embryo. Injected embryos are cultured to the tailbud stage and assayed for *globin* expression by whole mount *in situ* hybridization or Northern analysis. (D) *In situ* hybridization analysis of *globin* expression (purple stain) in embryos injected with increasing doses of FOG MO (*n = 4*). Arrowheads indicate the anterior-most aspect of the VBI, derived from dorsal cells not targeted by injections. (E) Northern analysis of *globin* expression in embryos injected with increasing doses of FOG MO (*n*≥*3*). (F) Northern analysis of *globin* expression in embryos injected with FOG MO (40 ng), xFOGr RNA (200 pg), or both (*n*≥*4*). Levels of *globin* expression are normalized to expression of the housekeeping gene *ODC* and reported as a percentage of control in the graphs in panel E and F. Error bars reflect S.D. Paired t test results are as follows: **, p≤*0.005;* ***, *p*≤*0.0005.*

To ask whether FOG is required for primitive erythropoiesis in *Xenopus*, we isolated a full-length *Xenopus* FOG cDNA (for cloning details, please see Material and Methods and Figure S1). We then used this sequence to design anti-sense MOs (illustrated in Figure 1A) capable of blocking translation of endogenous *x*FOG RNA. RNA encoding MYC-epitope tagged xFOG (xFOG-MYC) was injected alone, or together with FOG MO into *Xenopus* embryos at the two-cell stage, and expression of xFOG was examined by Western analysis of neurula stage (stage 15) embryonic extracts using antibodies specific for the MYC tag. Levels of xFOG-MYC protein translated from wild type RNA were reduced in the presence of the MO, whereas there was no effect on translation of a rescue RNA (xFOGr-MYC) containing silent mutations that prevent MO annealing (Figure 1B), demonstrating that the MO specifically targets wild type xFOG but not the xFOGr rescue RNA. FOG MO was then injected into both ventral vegetal blastomeres of eight-cell embryos (illustrated in Figure 1C) in order to target cells that are fated to give rise to the majority of blood-forming mesoderm [3], [42], [43]. Following injection, embryos were cultured to the tailbud stage (stage 34–36) and examined for changes in expression of the RBC differentiation marker *globin,* by whole-mount *in situ* hybridization. MO-mediated knockdown of xFOG resulted in a dose-dependent reduction in *globin* expression in the ventral blood island (VBI) (Figure 1D), which was further confirmed by Northern blot analysis (Figure 1E). *Globin* staining was preserved in the extreme anterior portion of the VBI in most embryos (Figure 1D, arrowheads), consistent with the fact that the anterior VBI is derived from cells originating on the dorsal side of the embryo, which were not targeted in our injections. We observed a significant reduction in *globin* expression with both 40 ng and 60 ng of FOG MO (Figure 1D, E), and all subsequent experiments were performed using the lower dose. Expression of the erythroid marker, *GATA-1,* was also reduced in FOG morphants (data not shown).

To demonstrate the specificity of our FOG MO for its target, we attempted to rescue expression of *globin* in FOG morphants by co-injection of xFOGr RNA, which does not anneal to the MO. Given prior studies demonstrating that overexpression of FOG can cause loss of blood [29], the quantity of xFOGr RNA was carefully titrated to achieve a dose that did not affect *globin* expression when injected alone. We found that injection of 200 pg of xFOGr RNA alone did not significantly effect *globin* expression (Figure 1F, lane 3), but was sufficient for rescue when injected together with FOG MO (Figure 1F, lane 4 compared with lane 2). These data demonstrate that xFOG is required for primitive erythropoiesis and are consistent with the hypothesis that FOG function in *Xenopus* is conserved with respect to other vertebrates in this context.

### Overexpression of FOG inhibits erythropoiesis independent of interaction with CtBP, NuRD, or GATA proteins

**Figure 2:**
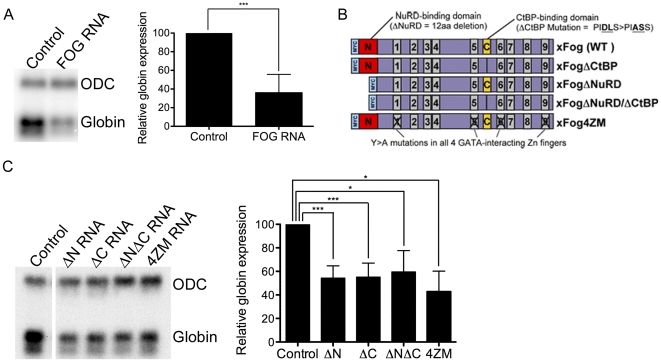
Overexpression of both wild type and mutant forms of xFOG inhibit primitive erythropoiesis. (A) Northern blot of *globin* expression in tailbud stage embryos in which wildtype xFOG RNA was injected into the VMZ of eight-cell embryos (*n = 8*). (B) Schematic of MYC epitope tagged wild type and mutant xFOG isoforms. The MYC tag is in blue, the NuRD binding domain in red, the CtBP binding domain in yellow and the zinc fingers are in grey. (C) Northern blot of *globin* expression in tailbud stage embryos that overexpress mutant forms of xFOG RNA injected into the VMZ of eight-cell embryos (*n*≥*4*). Levels of *globin* expression are normalized to expression of *ODC* and reported as a percentage of control. Error bars reflect S.D. Paired t test results are as follows: ***, p≤*0.0005;* *, *p*≤*0.05.* ΔN = xFOGΔNuRD, ΔC = xFOGΔCtBP, ΔNΔC = xFOGΔNuRD/ΔCtBP, 4ZM = xFOG4ZM.

To begin to address the discrepancy between the current results and previous studies in which overexpressed mFOG-2 or a truncated xFOG were shown to repress RBC development, we first asked whether overexpression of full-length *Xenopus* FOG also resulted in loss of RBCs. We injected RNA encoding wild type xFOG into the two ventral vegetal blastomeres of eight-cell embryos and analyzed expression of *globin* by Northern blotting (Figure 2A). Injection of 500 pg or more of wild type xFOG (doses comparable to those used in prior studies [29]) inhibited expression of *globin* (Figure 2A). While this was somewhat surprising given that FOG depletion also causes loss of blood in *Xenopus* (Figure 1), there are several explanations for this outcome. First, overexpression of mFOG-2 that cannot bind CtBP (mFOG-2ΔCtBP) in *Xenopus* embryos enhances RBC development [29], raising the question of whether endogenous xFOG might play a paradoxical role in repressing erythropoiesis, possibly by repressing genes required for erythrocyte maturation via CtBP recruitment [29]. Second, more recent studies have shown that downregulation of FOG is essential for commitment to certain non-erythroid hematopoietic lineages (e.g. eosinophil and mast cell lineages), and that prolonged or ectopic expression can disrupt FOG-independent functions of *GATA* genes, thereby preventing differentiation of these cell types [44]. Thus, the loss of RBCs observed upon overexpression of FOG in *Xenopus* may indicate that FOG must be downregulated to allow for differentiation of erythroid cells, similar to what is observed for eosinophils or mast cells. Third, ectopic expression of FOG in inappropriate cell types may interfere with other FOG-independent GATA activities required for blood. Fourth, overexpressed FOG may be prematurely facilitating GATA-1/FOG-1 induced repression at genes required for RBC progenitor expansion such as *GATA-2* [45], [46] and *Kit* [22], and thereby limiting the number of precursors available to differentiate as RBCs. Finally, it is possible that overexpression-induced loss of blood is a secondary consequence of deregulating the proper balance between FOG and its other binding partners. As such, overexpressed FOG may repress blood by binding and sequestering interaction partners such as GATA-1-2, NuRD and/or CtBP away from endogenous targets. One example of this phenomenon is suggested by the observation that Mta3, a component of the NuRD complex required for primitive erythropoiesis, appears to be recruited to hematopoietic targets through its association with FOG [47]. In this scenario, overexpressed FOG could block Mta3 function by sequestering it and other components of the NuRD complex away from target gene promoters, thereby inhibiting erythrocyte development. If this were the case, we reasoned that overexpression of FOG mutants in which these binding domains are abolished might fail to disrupt erythropoiesis.

To begin to distinguish among these possibilities, we generated several mutant forms of xFOG, including xFOGΔNuRD, xFOGΔCtBP, xFOGΔNuRD/ΔCtBP, and xFOG4ZM (as illustrated in Figure 2B and described in Materials and Methods). These mutations have been shown by others to prevent interaction with CtBP [23], the NuRD repressor complex [34], and/or GATA factors [11]. Mutant proteins were expressed at equivalent steady state levels in HEK cells, demonstrating that differences in activity are not likely to be due to differences in expression or stability (Figure S2). Impairment of GATA-2 binding was shown for xFOG4ZM, as loss of this particular interaction had not been previously verified (Figure S3). 500 pg of each xFOG mutant RNA was injected into the VMZ of embryos at the eight-cell stage as described above for WT xFOG, and *globin* expression was analyzed by Northern blotting at the tailbud stage. Surprisingly, overexpression of any of the mutant constructs produced a reduction in *globin* expression comparable to that seen with WT xFOG (Figure 2C).

Given that xFOG4ZM, which does not interact with GATA-1 or -2, is able to repress blood when overexpressed, it is unlikely that xFOG interferes with endogenous GATA-driven differentiation or progenitor expansion. Furthermore, as overexpression of FOG lacking either the CtBP or NuRD binding domain, or both caused a reduction in *globin* equivalent to that produced by overexpression of wild type FOG, abnormal repression of erythroid-specific genes is unlikely to account for the loss of blood. Instead, our results are most consistent with the possibility that overexpression of FOG leads to a dominant-negative squelching effect by which limiting amounts of transcriptional binding partners are prevented from accessing their endogenous target promoters. The discrepancy between our results showing that overexpressed xFOGΔCtBP represses erythropoiesis, and those of the previous studies showing that mFOG-2ΔCtBP enhances erythropoiesis might be due to species- and sequence-specific differences between xFOG and mFOG-2, or might be explained by the different methods used to evaluate erythropoiesis in the two studies. The observation that FOG deletion mutants that are unable to bind select co-factors are still able to repress erythropoiesis suggests that sequestering any one FOG binding partner is sufficient to interfere with erythropoiesis. Collectively, these findings suggest that precise regulation of FOG levels in the embryo is crucial for normal blood development.

### CtBP binding is dispensable, whereas NuRD binding is required for FOG function during primitive erythropoiesis

**Figure 3:**
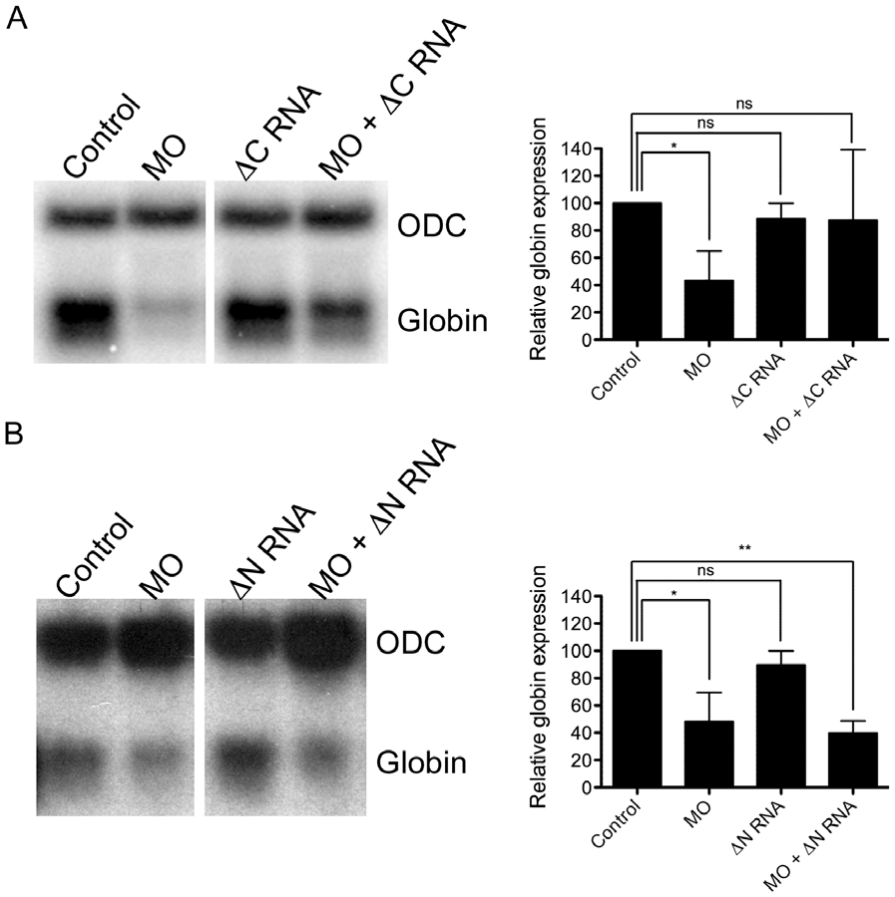
The NuRD binding domain of xFOG is essential for primitive erythropoiesis whereas the CtBP binding motif is dispensable. FOG MO (40 ng) and 200 pg of xFOGΔCtBP RNA (A) or xFOGΔNuRD RNA (B) were injected alone or together into the ventral vegetal blastomeres of eight-cell embryos and expression of *globin* was analyzed by Northern blotting at stage 34 (*n = 3 for each*). Levels of *globin* expression are normalized to expression of *ODC* and reported as a percentage of control. Error bars reflect S.D. Paired t test results are as follows: **, p≤*0.01;* *, *p*≤*0.05.* (ΔN = xFOGΔNuRD, ΔC = xFOGΔCtBP).

To better understand the relevance of FOG interaction with CtBP and NuRD in *Xenopus* primitive erythropoiesis, we tested the ability of each FOG mutant to rescue loss of blood in FOG morphants. xFOGΔCtBP contains silent mutations that prevent annealing of the FOG MOs and xFOGΔNuRD lacks the MO recognition sequence altogether due to its N-terminal deletion, thus both are able to serve as MO rescue constructs. To ask whether direct binding of CtBP is required for FOG function in primitive hematopoiesis in *Xenopus*, FOG MO was injected into the two ventral vegetal blastomeres of eight-cell embryos either alone, or together with 200 pg xFOGΔCtBP RNA and expression of *globin* was analyzed at the tailbud stage (Figure 3A). FOG morphant embryos showed reduced *globin* expression. However, co-injection of 200 pg of RNA encoding xFOGΔCtBP with the FOG MO significantly restored *globin* expression (Figure 3A). These studies, together with published studies showing that mFOG-1ΔCtBP knock in mice display no hematopoietic defects, suggest that direct recruitment of CtBP to FOG is not required for normal primitive erythropoiesis in vertebrates. However, it remains possible that CtBP is indirectly recruited to FOG by other nucleating factors that can themselves bind FOG. Precedence for this type of cooperative interaction between multiple binding partners, in which elimination of a single binding site does not interfere with functional complex assembly *in vivo* exists, for example, in the case of the axin destruction complex during Wnt signal transduction [48]. Chromatin immunoprecipitation to ascertain whether or not CtBP occupancy changes at blood-specific genes regulated by FOG-1 and GATA-1 or -2 in CtBP binding mutants (e.g. in FOG morphant embryos rescued with xFOGΔCtBP or in the *mFOG-1ΔCtBP* mutant mouse) relative to wild type controls would be necessary to definitively address this question *in vivo*.

In contrast to the ability of xFOGΔCtBP to substitute for wild type FOG during *Xenopus* primitive hematopoiesis, xFOGΔNuRD was not able to rescue loss of *globin* in FOG morphants in multiple experiments (Figure 3B). In some respects, this was unexpected given previous studies in which mutant forms of FOG lacking large portions of the N-terminus encompassing and extending beyond the NuRD binding domain are able to rescue erythroid differentiation in a *FOG-1* null cell line [35]. It is likely that functional differences between cell lines and *in vivo* differentiation, and the degree of overexpression attained in cell culture account for this discrepancy. Consistent with our findings, however, previous studies have shown that mice harboring knock-in alleles of FOG deficient in NuRD binding exhibit mild defects in definitive erythropoiesis and may have impaired primitive erythropoiesis as well [30], [31].

### Circulating RBCs in FOG morphant embryos are reduced in number and have abnormal morphology

**Figure 4:**
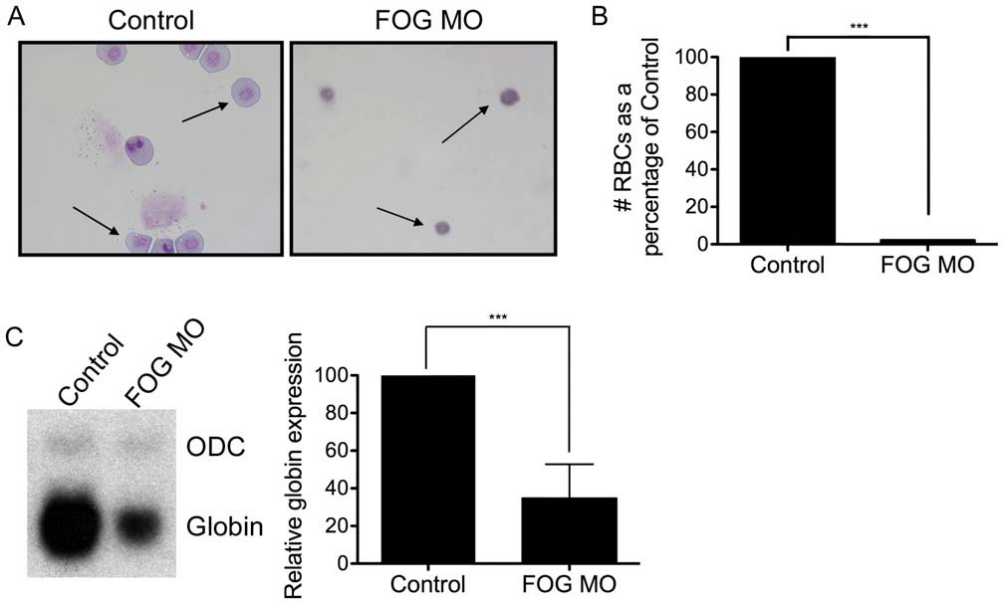
FOG morphants show a loss of circulating RBCs and altered RBC morphology. (A) Wright-Giemsa stain of blood cells from wild type and FOG MO injected embryos. Black arrows indicate primitive RBCs. (B) Graph showing the number of circulating RBCs in tadpoles injected with FOG MO at the eight-cell stage as compared to their wild type siblings. The number of RBCs is expressed as a percentage of control (*n = 4*). (C) Northern analysis of *globin* expression in stage 42 uninjected control and FOG MO injected embryos. Levels of *globin* expression are normalized relative to expression of *ODC* and reported as a percentage of control (*n = 6*). *Error bars reflect S.D. Paired t test results are as follows:* ***, *p*≤*0.0005.*

Our initial studies demonstrate that FOG is essential for primitive erythropoiesis in *Xenopus*, consistent with its role in other vertebrates. However, how it affects the cellular phenotype during this process is unclear. To begin to address this question, we first wished to determine whether xFOG was affecting erythroid development prior to differentiation. Consistent with previous data in fish showing that FOG is not required for specification of primitive blood [14], we found that expression of the RBC specification marker *Amyeloid Leukemia* (*Aml*) was unchanged as analyzed by *in situ* hybridization in stage 15 FOG morphants (not shown). We therefore looked to identify erythroid defects at later stages. To test whether xFOG affects primitive RBC differentiation or maintenance later in erythroid development, we collected total circulating blood cells from individual embryos at the tadpole stage (stage 42) and examined them for changes in RBC number and morphology. Compared to wild type sibling controls, the overall number of RBCs in FOG MO-injected embryos was drastically reduced (Figure 4A,B). In addition, FOG morphant RBCs were smaller in size, showed reduced cytoplasm and condensed nuclei relative to control RBCs (Figure 4A, black arrows). The nearly complete absence of circulating RBCs in FOG morphants was striking, given the more modest reduction in levels of *globin* at earlier (tailbud, stage 34–36) stages of development (Figure 1). However, when *globin* levels were analyzed by Northern blotting in FOG morphants at the tadpole stage (stage 42), we observed a more severe reduction in *globin* expression that paralleled the dramatic loss of circulating RBCs (Figure 4C). Together, our data indicate that depletion of xFOG in mesoderm leads to a loss *globin* at the tailbud stage. Moreover, this deficiency becomes progressively worse, and is reflected in a loss of red blood cells over time, suggesting that xFOG may be important in supporting erythrocyte survival. In support of this hypothesis, the morphology of the RBCs isolated from FOG morphants appeared consistent with that of cells undergoing programmed cell death, a phenotype that was not apparent in RBCs from wild-type siblings.

### Circulating RBCs in FOG depleted embryos show an increase in apoptosis

**Figure 5:**
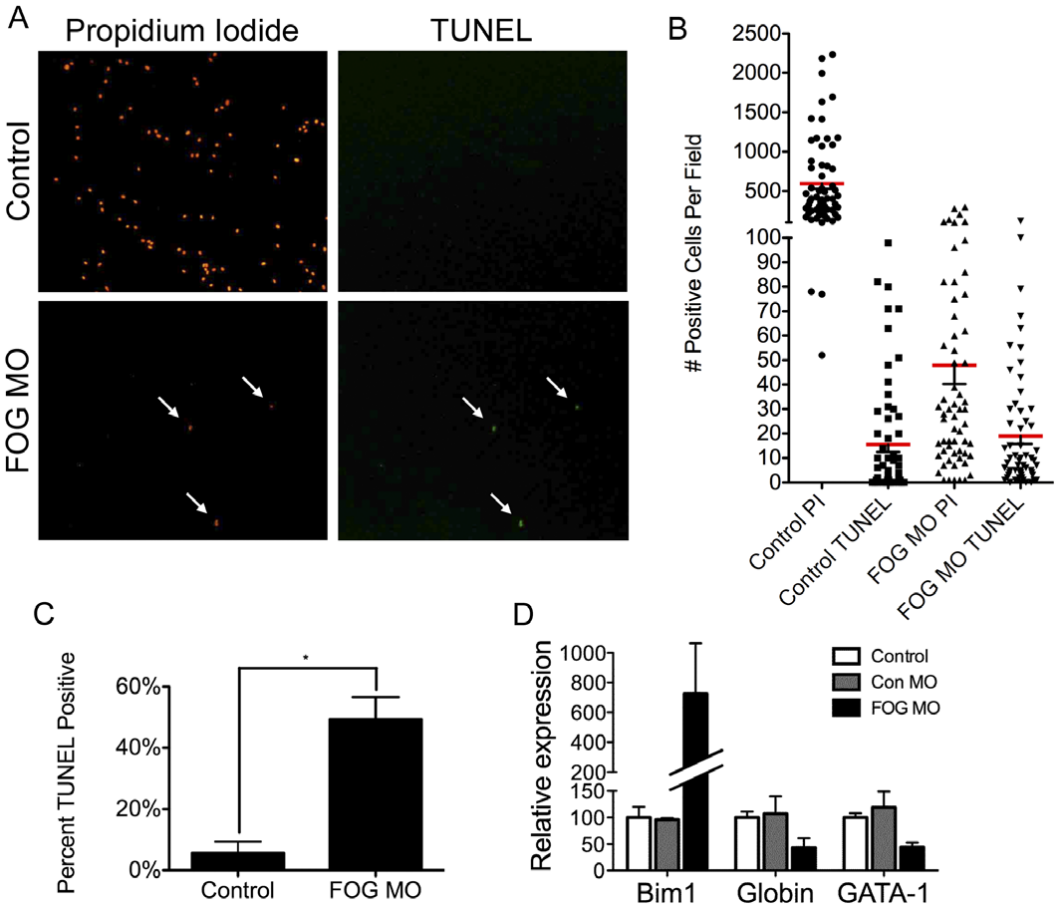
Loss of circulating RBCs in FOG morphants is due to increased apoptosis. (A) TUNEL staining of circulating blood cells. White arrows indicate cells that are both propidium iodide and TUNEL positive. (B) Scatter plot showing the distribution of numbers of propidium iodide (PI) and TUNEL positive cells per high-power field in blood from uninjected control and FOG MO injected embryos. Red bars indicate the mean (*n = 3*). (C) Graph of the number of TUNEL positive cells as a percentage of total (propidium iodide positive) cells in uninjected control and FOG MO injected embryos. (D) qPCR measuring expression of *Bim1*, *globin* and *GATA-1* in FOG MO injected embryos at the tailbud stage. Expression is normalized to expression of *ODC* and reported as percent of uninjected control *(n = 3). Error bars reflect S.D. for both Northern and qPCR analyses. Paired t test results are as follows:* *, *p*≤*0.05.*

To more stringently test the possibility that loss of xFOG results in excess erythrocyte apoptosis, we performed TUNEL assays on total circulating blood cells from wild type and FOG morphant tadpoles. We observed a significant increase in the fraction of TUNEL positive cells in FOG morphants (Figure 5A and B, quantified in Figure 5C), suggesting that FOG is required for survival of primitive erythrocytes, and that in the absence of FOG, these cells undergo excess apoptosis.

We also examined expression of the hematopoietic specific gene, *GATA-1*, and the pro-apoptotic gene, *Bim1* in tailbud stage embryos. *Bim1* is strongly upregulated in FOG morphants as early as the tailbud stage (stage 34), whereas levels of *GATA-1* are decreased (Figure 5D). GATA-1 is required for activation of most or all erythroid-specific genes, including those involved in mediating RBC survival [49], [50]. Specifically GATA-1 is known to directly activate expression of Lrf (also known as *Zbtb7a*/POKEMON/FBI-1), which in turn represses transcription of the pro-apoptotic gene, *Bim* [49]. Mice that harbor a targeted deletion of Lrf show increased apoptosis during terminal erythroid differentiation that results in a lethal anemia [49]. In a similar fashion, RBCs in FOG morphant embryos are able to form but then subsequently appear to recede and undergo a disproportionate degree of apoptosis compared to their wild type counterparts. Our data, which show that levels of *GATA-1* transcripts are reduced in FOG, morphants may reflect a requirement for FOG in maintenance or upregulation of *GATA-1* gene expression as development proceeds. Alternatively, given that regulation of most GATA-1-dependent genes requires binding to FOG, the loss of primitive erythropoiesis in FOG morphants may be due to loss of GATA-1 function, rather than expression, and this leads to upregulation of *Bim1* expression and excessive apoptosis of primitive RBCs. In this scenario, the reduction in levels of *globin* and *GATA-1* transcripts would be due to loss of RBCs rather than loss of transcription. Taken together, our data provide evidence for a model in which FOG regulates GATA-1 function and/or expression, which in turn directly or indirectly represses expression of pro-apoptotic genes such as *Bim* in order to support RBC survival.

## Supporting Information

Figure S1 **Alignment of** ***Xenopus*** **FOG with human, mouse and fish FOG homologs.** Alignment of full-length *X. laevis* FOG with zebrafish, mouse and human FOG-1 [51]. The NuRD binding domain is highlighted in red, the CtBP binding domain in yellow, the conserved zinc fingers in gray and a putative tenth zinc finger conserved in fish and frogs is highlighted in green.
(DOC)

Figure S2 **Mutations in xFOG do not affect steady state levels of FOG protein. **Western analysis of cell lysates from HeLa cells transiently transfected with MYC epitope tagged wild type and mutant xFOG cDNA constructs. Steady state levels of xFOG protein are normalized to expression of Actin and reported as a percentage of the wild type control below each lane. Asterisk indicates a non-specific background band that is observed in the absence of xFOG. ΔN = xFOGΔNuRD, ΔC = xFOGΔCtBP, ΔNΔC = xFOGΔNuRD/ΔCtBP, 4ZM = xFOG4ZM.
(TIF)

Figure S3 **GATA-2 binding is severely impaired in the xFOG4ZM mutant. **Wild type and mutant MYC epitope tagged xFOG constructs were transfected into HeLa cells alone, or together with HA-tagged GATA-2. Western blots (WB) of cell lysates (input) or anti-MYC immunoprecipitates (IP) were probed with an anti-HA antibody to detect interaction with GATA-2-HA. Levels of GATA-2 protein detected by co-IP are normalized to the GATA-2 input and to the xFOG IP and reported as a percentage of the wild type control below each lane. ΔN = xFOGΔNuRD, ΔC = xFOGΔCtBP, ΔNΔC = xFOGΔNuRD/ΔCtBP, 4ZM = xFOG4ZM.
(TIF)
