# Supplementary material for: Friend of GATA (FOG) Interacts with the Nucleosome Remodeling and Deacetylase Complex (NuRD) to Support Primitive Erythropoiesis in Xenopus laevis
Source: PLoS One. 2012 Jan 3;7(1):e29882. doi: 10.1371/journal.pone.0029882 (PMC3250481; doi:10.1371/journal.pone.0029882)
Supplement: Figure S1 — Alignment of Xenopus FOG with human, mouse and fish FOG homologs. Alignment of full-length X. laevis FOG with zebrafish, mouse and human FOG-1 [51]. The NuRD binding domain is highlighted in red, the CtBP binding domain in yellow, the conserved zinc fingers in gray and a putative tenth zinc finger conserved in fish and frogs is highlighted in green. (DOC) [file pone.0029882.s001.doc]

**Figure S1**

**Z MSRRKQSKPR QIKRSIGDLN GGEDPS.DDV SMSGEEGGAS DQEDSAECDG SSPHSFTP..**

**X MSRRKQSNPR QIKRSLGDME GTEAKFIEEP NHSDKDGAYS DQDGSVDCDS PSPVNSDSNE**

**M MSRRKQSNPR QIKRSLRDME AGEEAKAMDS SPK..EQEAP DPEAPAIEEP PSPPREDVSP**

**H MSRRKQSNPR QIKRSLGDME AREEVQLVGA SHM..EQKAT APEA...... PSPPSADVNS**

**Z .......... .......... .......LYN EEPRTHESLA VSDEGEEDEK GLKRCTEDEE**

**X ENGCNSVTQS LEQESEEAAS KPSVELGQIT KSPCTSEGEL .....REDEE NIQESRSPSS**

**M .......... .......... .PAV...... PAPPESPEDP EDMEGQELEM RPQDEEKEEK**

**H .......... .......... .PPP...... LPPPTSPGGP KELEGQEPEP RP........**

**Z EEDVDREGEP QWNGPDDLVL SG..SSDDFK VLALRDLSGD TVWGPFSGSI QSGEPTDGPA**

**X T..EDAEEPQ IWNGPDELEL EISSTDGVGH IRARSQLHKG FSWGPYKGNF TGSSSSPSPA**

**M E..EEAAMAS PWSGPEELEL AL..QDGQRC VRARLSLTEG LSWGPFYGSI QTRALSPERE**

**H ...TEEEPGS PWSGPDELEP VV..QDGQRR IRARLSLATG LSWGPFHGSV QTRASSPRQA**

**Z SESSAVSLVC EEPDCWLRRI PVTSNPTDSN CTIYSQGGVL FCKLTRELSG GDALLASLSS**

**X DLSISLSLD. VDDDCWLKYM TLVSCEAEAN AVLYRKGDQI WCKTSQTVEQ GEVIQAFLMA**

**M EPGPAVTLM. VDESCWLRML PQVLTEEAAN SEIYRKDDAL WCRVTKVVPS GGLLYVRLVT**

**H EPSPALTLLL VDEACWLRTL PQALTEAEAN TEIHRKDDAL WCRVTKPVPA GGLLSVLLTA**

**Z SNGDHSAGTQ THGVRVKEEP AYP...AALH SEIQLLPQQA GMAAILATAV VNKDIFPCKD**

**X EP..Q..AIP NYTI..KEEP GETSQCTSTL PEFQLLPQQA GMAAILATAV VNKDVFPCKD**

**M EP..H..GAP RHPV..Q.EP VEPGGLAPVH TDIQLLPQQA GMASILATAV INKDVFPCKD**

**H EP..H..STP GHPV..KKEP AEPTCPAPA. HDLQLLPQQA GMASILATAV INKDVFPCKD**

**Z CGIWYRSERN LQAHLMYYCA SRQKQQTAA. SPPQDKPKDS YPNERLCPFP QCNKSCPSAS**

**X CGIWYRSERN LQAHLMYYCA SRQSSTSP.. .SMEEKPKDS YPNERICPFP QCKKSCPSSS**

**M CGIWYRSERN LQAHLLYYCA SRQRAGSPV. SATEEKPKET YPNERVCPFP QCRKSCPSAS**

**H CGIWYRSERN LQAHLLYYCA SRQGTGSPAA AATDEKPKET YPNERVCPFP QCRKSCPSAS**

**Z SLEIHMRTHS GERPFVCLIC LSAFTTKANC ERHLKVHTDS LNGVCHGCGF ISTTRDILYS**

**X SLEIHMRSHS GERPFVCLIC LSAFTTKANC ERHLKVHTDT LNGVCHGCGF ISTTRDILYS**

**M SLEIHMRSHS GERPFVCLIC LSAFTTKANC ERHLKVHTDT LSGVCHNCGF ISTTRDILYS**

**H SLEIHMRSHS GERPFVCLIC LSAFTTKANC ERHLKVHTDT LSGVCHSCGF ISTTRDILYS**

**Z HLVTSHMVCQ PGSNSEVYSP KLPVAAGLSP G..D....SG IVLKCQVCGY SADTPALLQ.**

**X HLVTNHMICQ PGSKVDVYPV VKAVPAVKSS NPVVSQIASS SLLKCGLCGF LADGLPSLQ.**

**M HLVTNHMVCQ PGSKGEIYSP GAGHPAAKLP .......... .......... .PDSLAGFQ.**

**H HLVTNHMVCQ PGSKGEIYSP GAGHPATKLP .......... .......... .PDSLGSFQQ**

**Z QHVHTHLEVR V.PAER..SP TPRQSSPPSS ELPELQETEP AACVPRPDSS SPG..ANGSS**

**X QHALLHTTNP VPSATHSV.K SPPENINEK. QNPE..SQ.. ....ENGNAK SPI..SSSSS**

**M QHSLMHS..P LVPADK..AP TPSSGLDSK. ..AE...V.. ....TNGETR VPP..QNGGS**

**H QHTALQG..P LASADLGLAP TPSPGLDRK. ALAE...A.. ....TNGEAR AEPLAQNGGS**

**Z ASQGYSPLS. QLNIKEEPRS DYENDAKEEE Q.VNSPQENA AEASSSQPTS PKSPTVVAVK**

**X ASSRSEETPL KLYIKQEPE. ........GQ LSISEAGSTT CEAKDGVA.L VQSPA.IKVK**

**M SESPAAPRTI KVEAAEEPE. ........AT R.ASGPGEPG PQAPSRTP.S PHSPNPVRVK**

**H SEPPAAPRSI KVEAVEEPE. ........AA P.ILGPGEPG PQAPSRTP.S PRSPAPARVK**

**Z AEPASPTPGS SPAHSGTGGS VLPGGAVFLP QYMFNSEAA. ....IMPQAS EILAKMSEMV**

**X TEMSSPTPGS SPVPNETGA. ATGGGTVIIP HYVFGHEAT. ..AAIVPQAS EILAKMSELV**

**M TELSSPTPGS SPGPGEL... .TMAGTLFLP QYVFSPDAGT TTVPTAPQAS EILAKMSELV**

**H AELSSPTPGS SPVPGEL... .GLAGALFLP QYVFGPDA.. .....APPAS EILAKMSELV**

**Z HSRLKQGQGP A.AAQQSFYP PGSPASVHKG ATCFECDITF NNINNFYVHK RLYCSSRHQQ**

**X HSRLKQGQAV T.PA...GFS ...GSAVPKG ATCFECEITF NNINNYYVHK RLYCSGRHVS**

**M HNRLQQGAGS SGAA...GTP TGLFSG.TKG ATCFECEITF NNINNFYVHK RLYCSGRRAP**

**H HSRLQQGAGA G.AG...GAQ TGLFPGAPKG ATCFECEITF SNVNNYYVHK RLYCSGRRAP**

**Z GETGGLVKEG AVTAAAPPAS HAASPQARPV SRAASASPSC PDPAPGG... .....TASEP**

**X DEN....... .......... .......SSS ARKVKAL.PA RTALASGFSS TEQEASPPQE**

**M .ED....... .......... .......PPT VRRPKAA.TG PARAPAG... .....AAAEP**

**H .ED....... .......... .......APA ARRPKAP.PG PARAPPG... .....QPAEP**

**Z KVVEVK.IED PGLKDATCSS SSEGEGPGGG QASEGSQSPS GSAEDQDDDP TRTFCQACNI**

**X DAGEESSAPV VAVKLEENSG .MDCEGAGSG HVSEGSQSPS .SLDDPEEDP NRTVCGACNI**

**M DPSRSS..PG PGPREEEASG TTTPEAEAAG RGSEGSQSPG SSVDDAEDDP SRTLCEACNI**

**H DAPRSS..PG PGAREEGAGG AATPEDGAGG RGSEGSQSPG SSVDDAEDDP SRTLCEACNI**

**Z RFSRHDNYIV HKRFYCASRH DPTNQRPHSG KA.......A F..LPQPIRT RKRKKMYEIH**

**X RFSRHETYVV HKRYYCASRH DPPLRRREVN K..PGP..PY T..TQPTPRT RKRRKLYEIH**

**M RFSRHETYTV HKRYYCASRH DPPPRRPPAP TTAPGPAAPA L..TAPPVRT RRRRKLYELP**

**H RFSRHETYTV HKRYYCASRH DPPPRRPAAP PGPPGPAAPP APSPAAPVRT RRRRKLYELH**

**Z MAQTEALANA TTLPLGTSLG ..VNQEG.SS VALLSQVSTP TRSSSP.EGE GPIDLSKRPR**

**X GVA.PTEST. ..PPSPHTLG R.VEAMA.LM PGLIPAPVMP SPSSSPDAVD GPIDLSKKPR**

**M AAGAPPPA.A GPAPVPVVPS PTAELPSSPR PGSASAGPAP ALSPSP.VPD GPIDLSKRPR**

**H AAGAPPPPPP GHAPAPESPR PGS.GSG... ...SGPGLAP ARSPGP.AAD GPIDLSKKPR**

**Z LRESQ.RKDS IS.ALPLSDY HKCTACSISF NSIENYLAHK TYYCPATTLQ PQTTEQLNRL**

**X LVAEAPVPSA AATVAPLADY HECTACRISF NSLESYLAHK KFSCPTAPLQ QKTIQQLQKV**

**M R....QSPDA PTALPALADY HECTACRVSF HSLEAYLAHK KYSCPAAPLR ..........**

**H R....PLPGA P..APALADY HECTACRVSF HSLEAYLAHK KYSCPAAPPP GA........**

**Z KRPASTSPKN RAVDQHSDSK V.....LQTG KTAA..VAHA VPGS.ESTPP HVQGA....K**

**X KSPSSATGK. .LVDDTVKVK VESKAALSPG SVSETIQPLA LPFSTISDPK QLQQYSSVTE**

**M .......... .......... .......... .......... .......... ..........**

**H .......... .......... .......... .......... .......... ..........**

**Z TPSTSPVVCP YCPPNKLLTC DLMEHFKTTH GLVLTLQQ.H PETQSTGV.. ...SPSPSLS**

**X ASLSATTTCP YCPHNVIIRG DLLEHFRSVH GLILAKPTAG HRLQTTFMEV LVPARGQTSS**

**M ....TTALCP YCPPNGRVRG DLVEHLRQAH GLQVAKPAAS PGAEPR.... .TPA......**

**H .LGLPAAACP YCPPNGPVRG DLLEHFRLAH GLLLGAPLAG PGVEAR.... .TPA......**

**Z PREGAPLTP. PK........ PSSRPRKDSL NGRRIKLEAT SPSPPVLNGS SLESVGSRSP**

**X ASENSLPSPP .VSSASPLQL PGLRRENSNF KDTT.SSSSS ANGSPILTST PRPLL...PT**

**M .E........ .......... ...RAPRDSP DG........ ..RAPRSPSP APENT...PS**

**H .D..RGPSPA PAPAASP..Q PGSRGPRDGL GPEP.Q..EP PPGPPPSPAA APEAV...PP**

**Z KTAPPALSPK GVTVSPVPEA LRETGQLSHT PLPTVLPEKA ALAISHTHTA PPKTPL....**

**X SPAPPSNS.. ....LPLAES RREDG.LPRV PSQVLLPGDK AM.......Q PPKPSL....**

**M DPA....... .......... .......... ........DQ GA.......R TPSKGPPAPA**

**H PPAPPSYS.. .......... .......... ........DK GV.......Q TPSKGT....**

**Z ASPLQNGNTR YCRLCNIKFS SLSTFIAHKK YYCSSHSAEH VK**

**X ISPVPNGNHR YCRLCNIKFS SLSTFIAHKK YYCSSHAAEH VK**

**M PAPGGGGGHR YCRLCNIRFS SLSTFIAHKK YYCSSHAAEH VK**

**H PAPLPNGNHR YCRLCNIKFS SLSTFIAHKK YYCSSHAAEH VK**
