# Supplementary figures and images for: Friend of GATA (FOG) Interacts with the Nucleosome Remodeling and Deacetylase Complex (NuRD) to Support Primitive Erythropoiesis in Xenopus laevis
Source: PLoS One. 2012 Jan 3;7(1):e29882. doi: 10.1371/journal.pone.0029882 (PMC3250481; doi:10.1371/journal.pone.0029882)

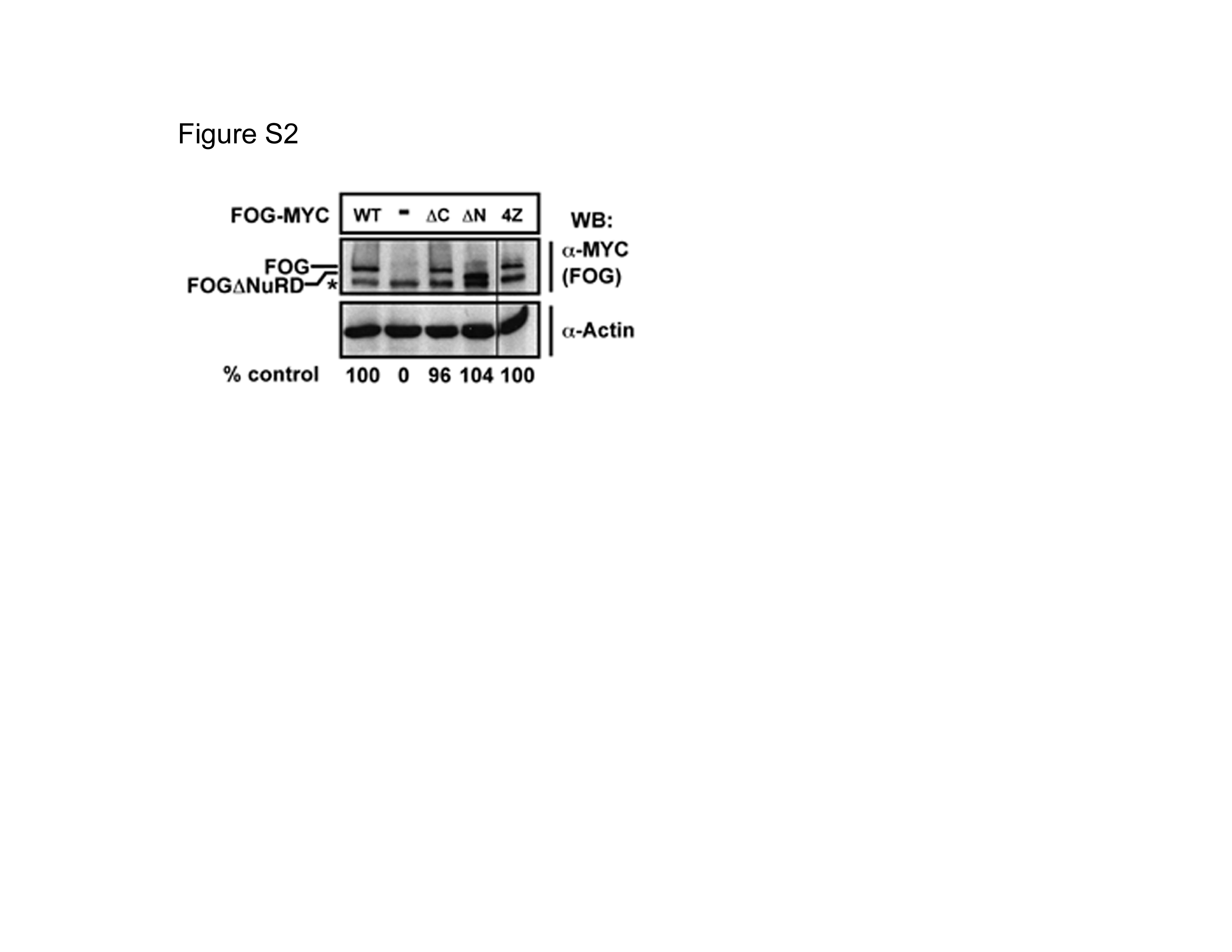

Supplement: Figure S2 — Mutations in xFOG do not affect steady state levels of FOG protein. Western analysis of cell lysates from HeLa cells transiently transfected with MYC epitope tagged wild type and mutant xFOG cDNA constructs. Steady state levels of xFOG protein are normalized to expression of Actin and reported as a percentage of the wild type control below each lane. Asterisk indicates a non-specific background band that is observed in the absence of xFOG. ΔN = xFOGΔNuRD, ΔC = xFOGΔCtBP, ΔNΔC = xFOGΔNuRD/ΔCtBP, 4ZM = xFOG4ZM. (TIF) [file pone.0029882.s002.tif]

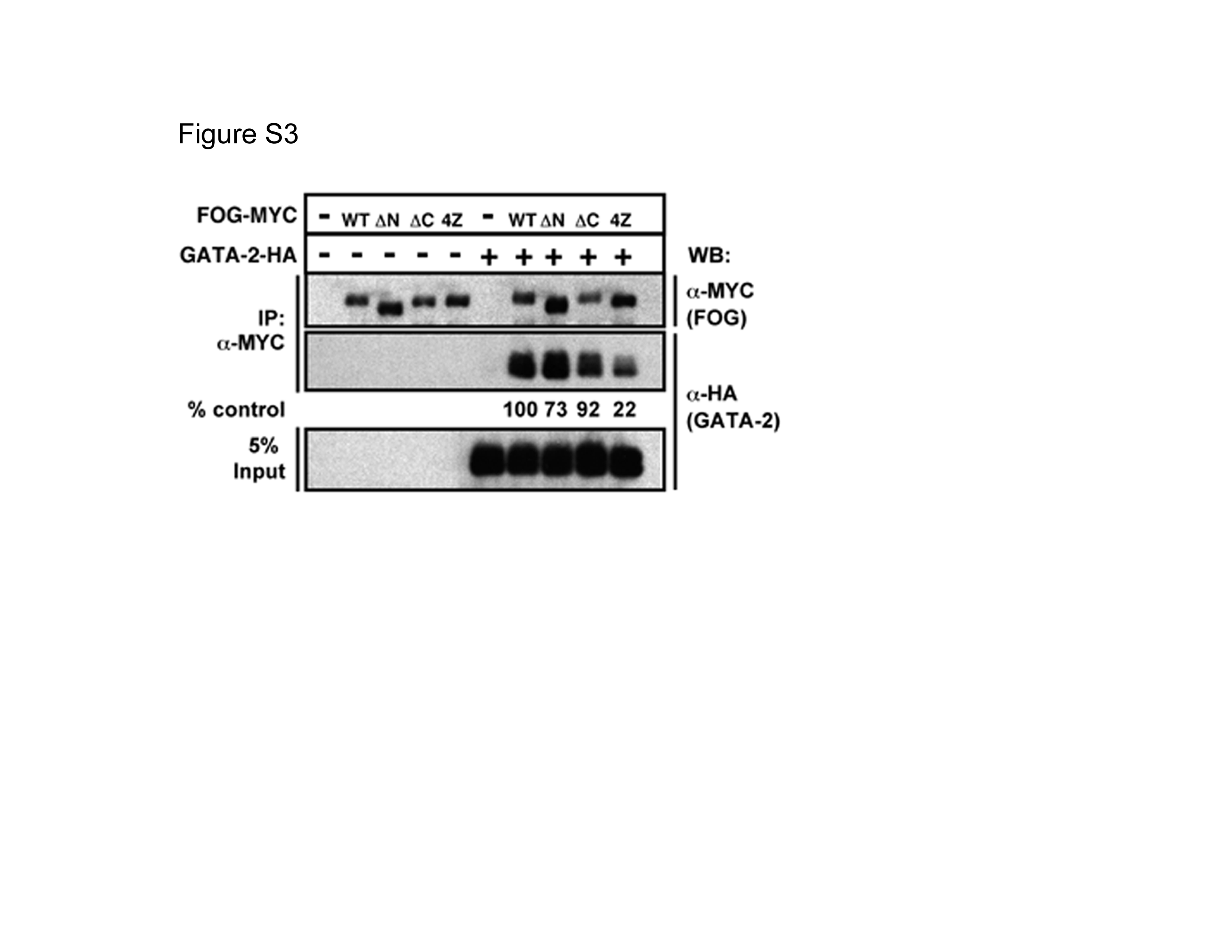

Supplement: Figure S3 — GATA-2 binding is severely impaired in the xFOG4ZM mutant. Wild type and mutant MYC epitope tagged xFOG constructs were transfected into HeLa cells alone, or together with HA-tagged GATA-2. Western blots (WB) of cell lysates (input) or anti-MYC immunoprecipitates (IP) were probed with an anti-HA antibody to detect interaction with GATA-2-HA. Levels of GATA-2 protein detected by co-IP are normalized to the GATA-2 input and to the xFOG IP and reported as a percentage of the wild type control below each lane. ΔN = xFOGΔNuRD, ΔC = xFOGΔCtBP, ΔNΔC = xFOGΔNuRD/ΔCtBP, 4ZM = xFOG4ZM. (TIF) [file pone.0029882.s003.tif]
